# Control on Surface Plasmon Polaritons Propagation Properties by Continuously Moving a Nanoparticle along a Silver Nanowire Waveguide

**DOI:** 10.1038/srep37512

**Published:** 2016-11-22

**Authors:** Fan Wu, Wenhui Wang, Jiaojiao Hua, Zhongfeng Xu, Fuli Li

**Affiliations:** 1 School of Science, Xi’an Jiaotong University, Xi’an 710049, China

## Abstract

Surface plasmon polaritons (SPPs)-based nanowire waveguides possess potential applications for nanophotonic circuits. Precise control on the propagation of SPPs in metal nanowires is thus of significant importance. In this work, we report the control on SPPs propagation properties by moving a silver nanoparticle (Ag NP) along a silver nanowire (Ag NW). The emission intensity at NP can be attenuated to about 25% of the maximum emission value with increasing the distance between excitation end and NP. When NP is gradually moved away from excitation end, the intensity of emission light at Ag NP shows an exponential decay with a superposition of wavy appearance, while the emission at NW end is almost a constant value. It is found that the former is related to the local SPPs field distribution in NW, and the latter is dependent on the distance between excitation end and NW terminal. Moreover, the propagation loss in Ag NP-NW structure has been investigated. Our experiments demonstrate the important role of NP location in NW-based waveguides and provide an effective method of tuning scattering light in NW, which is instructive to design the future specialized function of SPPs-based nanophotonic circuits and devices.

Electronic integrated circuits are currently manufactured with decreasing the dimensions of components below 100 nm, resulting in the knotty problems on interconnect delay and heat radiation, which seriously affect the reliability and increase the cost of electronic devices^1^,^2^. With the advantages of high speed and wide bandwidth, photonic circuits have great potential for replacing the traditional electronic devices in the future information technology^3^,^4^. However, the miniaturization of photonic devices is limited owing to the diffraction limit of light. Due to the unique ability of circumventing this limit and concentrating light into the deep sub-wavelength scale, surface plasmon polaritons (SPPs)-based nanophotonic circuits have attracted increasing attentions^5^,^6^,^7^,^8^,^9^. SPPs are light waves coupled to free electron oscillations in a metal, which exists at the metal-dielectric interfaces^10^,^11^,^12^. Metal nanowires (NWs) with good morphology and high crystallinity are excellent waveguides to support SPPs^13^,^14^,^15^,^16^. In NW-based nanophotonic devices, one of the significant issues is to precisely control the behaviours of SPPs propagation, especially the position where SPPs can be scattered into photons. This is essential for the performance of nanophotonic devices with special functions in NW-based waveguides, such as selective all-optical switch^17^,^18^,^19^, remote SERS excitation^20^,^21^,^22^,^23^, and light source^24^,^25^,^26^. For an individual NW without fabrication imperfections, SPPs can only be scattered into photons at the NW terminals, rather than the middle areas of NW. However, in order to realize some functions in NW-based nanophotonic devices, SPPs are required to be scattered as photons at the middle section of the NW. To accomplish this task, methods that can induce symmetry breaking are usually used. Generally, there are two routes to introduce the symmetry breaking in a NW: placing another wire or a NP nearby the NW^27^,^28^,^29^. For the former, the position of branch NW cannot be easily manipulated, while for the latter, the position can be flexibly tuned with more precision. This advantage enables NP-NW structure a promising application in SPP-based nanophotonic devices. Recently Pan *et al*.^29^ has theoretically predicted the significant influence of NP position in a branched NW on the performance of tunable switchers. By tuning NP location, the propagation behaviour of SPPs is controlled and results in the switch power between the two NW branches. The precise and flexible control over the NP position in NW-based waveguides is a significant and meaningful issue for the development of nanophotonic devices. However, the experiments on tuning the NP location along a chemically synthesized NW have not been reported and are still challenge.

In this work, we realize the control of SPPs propagation properties in Ag NW through continuously tuning the position of Ag NP along the NW. We investigate the change of the emission intensities at NP and NW end with tuning the NP position. The emission intensity at NP shows an exponential decay together with a wavy pattern when the NP is moved continuously away from the excitation end, while the emission at NW end is almost a constant value. We find that the emission intensity at NP is related to the strength of the local SPPs field in NW and the intensity at NW end is mainly dependent on the distance between NW terminal and excitation end. Moreover, instead of tuning the NP position, we investigate the emission behaviour in NP-NW geometry through changing the position of excitation, and study the change of propagation loss at different NP locations. Our results indicate the significant role of NP position in NW-based waveguides and shed light on the structural design of future nanophotonic devices.

## Materials And Methods

Chemically synthesized Ag NWs with smooth surfaces are good candidates to support SPPs^30^,^31^. In our experiments, Ag NWs and NPs were synthesized through a multi-step method^32^. The obtained products were washed with acetone, ethyl alcohol and deionized water for several cycles, and then the suspension was dropped on a cleaned glass substrate for subsequent manipulations.

A 785 nm semiconductor laser (DL785-070-SO, Crystal Laser) introduced by a tapered optical fiber^14^,^33^ was employed to excite the propagating SPPs in Ag NW waveguides. Another tapered optical fiber was used for applying a force to continuously move the NP (along the NW). Unless stated otherwise, the emitted intensity at NW end and the location of NP were collected by a 100 × objective (N.A. = 0.95, Olympus) and the optical images were collected by a high-sensitivity charge-coupled-device (CCD, DR-328G-C01-SIL, Andor) camera through an inverted optical microscope (IX73, Olympus). The emitted light intensities at the NP and Ag NW terminal were directly obtained by determining the maximum value in the output spot. The position of NP was precisely controlled by three-axis translation stage and directly measured from bright-field optical image.

The finite-difference time-domain (FDTD) simulation (Lumerical Solution) of the experimental structures is applied to model the system. Mesh size of 3 nm and boundary of perfectly matched layers (PML) are found accurate enough for the simulations. The optical constants of Ag were adopted from the experimental data by Johnson and Christy^34^. The polarization angle of excitation Gaussian beam (wavelength 632.8 nm) was 45° towards the nanowire axis. The beam width is 0.6 μm. The gap between the NP (nanosphere, *D*_*S*_ = 100 nm) and the main NW (*L*_*W*_ = 20.3 *μ*m, *D*_*W*_ = 300 nm) is 3 nm.

## Results and Discussion

**Figure 1:**
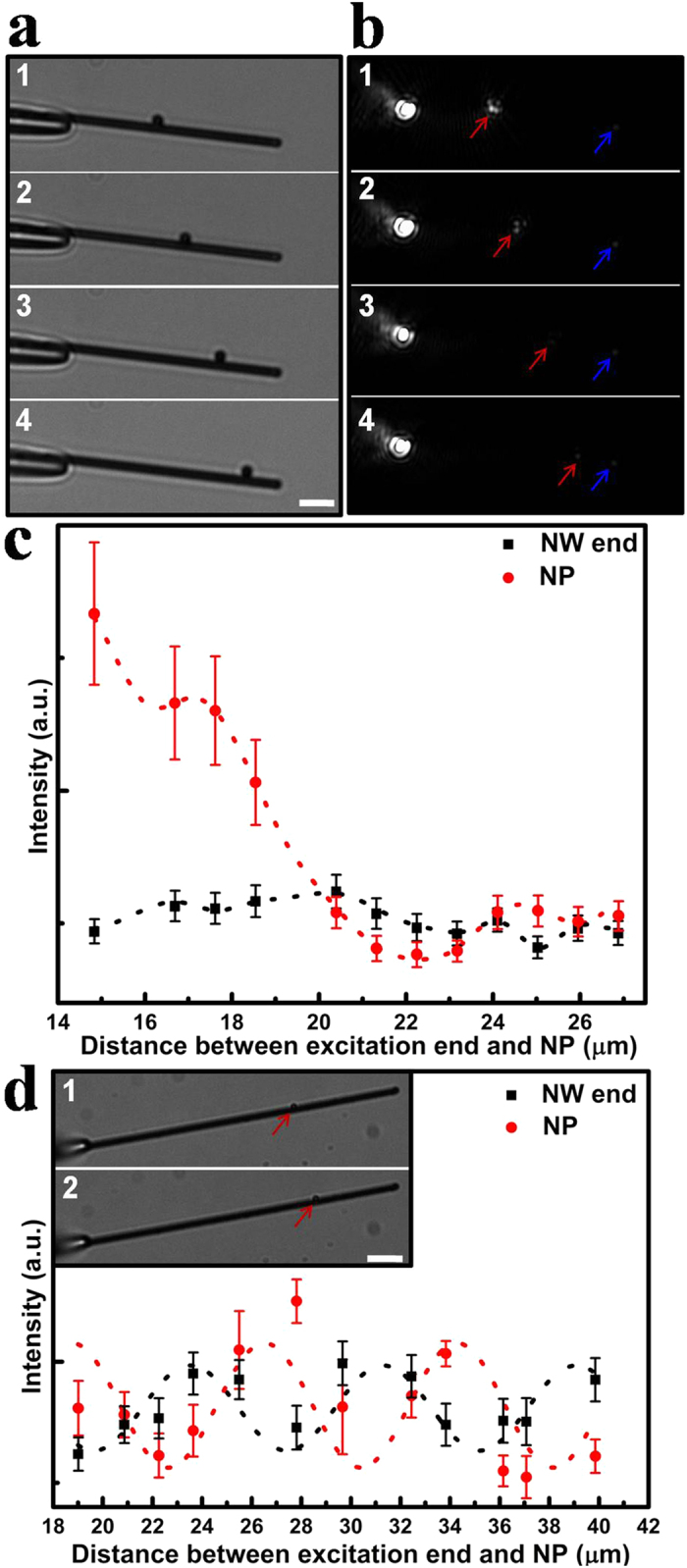
Characterization of emissions at an Ag NP-NW waveguide. (**a**) Bright-field optical images collected by a 50× objective (N.A. = 0.80, Olympus). (**b**) The corresponding dark-field optical images. Emission spots at Ag NP and NW end are indicated by red and blue arrows respectively. (**c**) The emission intensities at NP and NW end for different distances between NP and excitation end. Dotted curves correspond to the fitted emission intensity. (**d**) Emissions at another Ag NP-NW waveguide. The inset shows the selected bright-field optical images of Ag NW with diameter of 750 nm and length of 46 μm, and Ag NP with diameter of 630 nm, and the red arrows indicate the positions of NP. The scale bar is 5 μm. The error bars show the standard deviations of emission intensities from data collected within about 2 μm ranges.

Ag NWs with diameters ranging from 600 to 1000 nm and lengths from 30 to 50 μm, and Ag NPs with size of 600 to 1200 nm were employed in our experiments. The light emissions at NP and NW end are collected and characterized by an inverted optical microscope. Selected bright field optical images illustrated in Fig. 1a demonstrate the process of continuously moving Ag NP with edge length of 1.2 μm along an Ag NW with length of up to 35 μm. The corresponding dark-field images are shown in Fig. 1b. The scattered lights at the position of NP and NW end can be clearly observed. The several bright points appearing at NP in picture 1 and 2 is probably related to the scattering at sharp corners in the irregular-shaped NP. The emissions at NP and NW end are quantitatively shown at Fig. 1c. The intensity at NP is attenuated to about 25% of the maximum emission value with increasing the distance from 15 μm to 27 μm. It is interesting to note that the emission intensity at NP shows an exponential decay trend with an undulating pattern, while the intensity at NW end is almost a constant value when NP is gradually moved from the excitation end to NW end. To analysis the mechanism of the emission behaviour, we should refer to the situation in an individual NW without NP. As is well known, the emission tendency at NW end is exponential decay, which arises from the intrinsic Ohmic loss in metal NW^14^,^35^. The SPPs field distribution can directly reflect the Ohmic loss in NW, which shows an exponential decay with increasing the propagation distance. In NP-NW structure, the propagating SPPs can be scattered out as photons at the position of NP, because the momentum mismatch between light and SPPs can also be compensated at NP through scattering process. As scattering intensity is proportional to strength of local SPPs field, the emission illustrates the exponential decay trend. This exponential damping tendency mainly appears at the distance below 20 μm. When the distance between excitation end and NP is over 20 μm, there is no apparent emission damping. As the propagation distance increased, more proportion of SPPs energy has been lost due to the Ohmic loss in NW, which results in the relative slow decay. In addition, the wavy behaviour in the emission intensity probably results from the beat SPPs field distribution in NW. Specifically, for a glass-supported Ag NW, SPP modes with different azimuthal symmetries can be excited when the fiber is placed close enough to Ag NW^36^,^37^. The interference between excited SPP modes and the counter propagating SPP modes resulting from reflections by the terminals of NW^37^,^38^ can form the beat SPPs field distribution in NW. For this beat distribution, SPPs field strength is strong at the antinode while weak at the node. When the NP is placed at an antinode, the strong SPPs field induces relative strong emission light at NP. On the contrary, when the NP is placed near the node, the emission at NP becomes weak. With continuously moving, NP can alternately experience the node and antinode, leading to the wavy pattern with a decreasing trend in the emission at NP.

As far as the end of NW is concerned, the emission intensity is almost a constant value, which is very different from that of an individual NW as previously reported^35^. As the distance between the excitation end and NW end in the case of single NW is continuously changed, the corresponding Ohmic loss will be changed. In contrast, the distance in Fig. 1c is kept constant and the Ohmic loss is a constant, which would account for the nearly unchanged emitted intensity at NW end. Although the emission intensity at NW end mainly depends on the distance between excitation end and NW end, the fluctuation at NW end emission intensity can be probably ascribed to the existence of NP, which can affect the emission at NW end to a certain extent.

When NP is placed away from the excitation end, emissions at NP and NW end show a roughly opposite oscillation. This phenomenon is more obvious in an Ag NP-NW structure with longer propagation distance, as shown in Fig. 1d. When NP is set at the antinode, more SPPs are scattered into photons at the NP location, which results in the relative weak emission at the NW end, and *vice versa*. The period of this wavy pattern is about 7 μm, which can roughly reflect the period of SPPs field distribution along the wire. As previously reported^37^, the period of SPPs field distribution is about 2~3 μm displayed by the quantum dot fluorescence technique. However, the oscillating period presented here is larger. This can be mainly attributed to the thick and long NWs employed in our experiments, which results in the relative large period of SPPs field distribution. The display precision of the period is also probably dependent on the step length when moving NP in the experiments. In addition, the oscillating trend of emission intensities is related to the morphology (shape and size) of NP-NW geometry, and a more detailed investigation of the influence of the morphology on the SPPs scattered out from NW-based waveguides is in progress.

**Figure 2:**
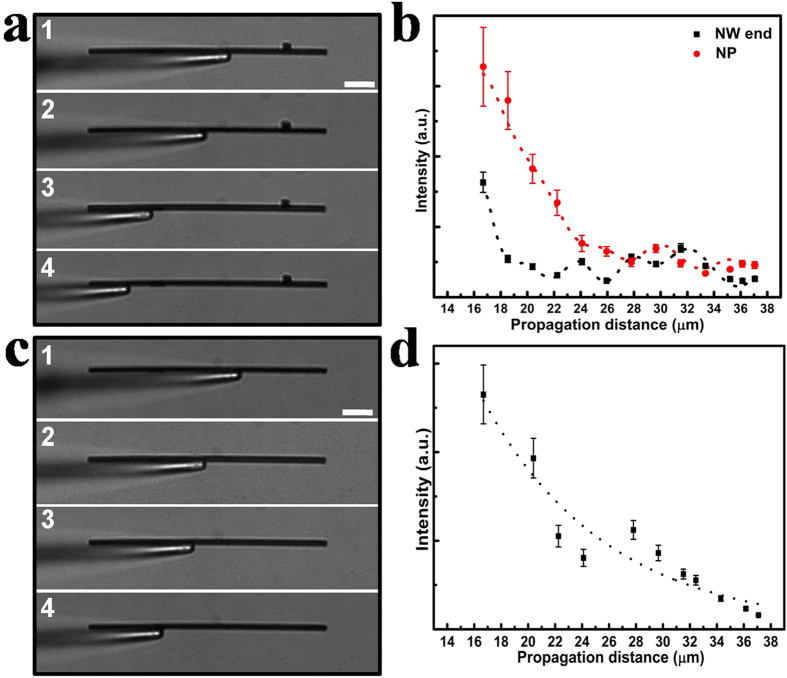
Emissions in Ag NW waveguide with and without NP. (**a**) Bright-field optical images of Ag NP-NW structure. (**b**) The correponding emission intensities at Ag NW end and NP for different propagation distances. Dotted curves are the fitted emission intensities at NP and NW end respectively. (**c**) Bright-field optical images of the Ag NW without Ag NP (this Ag NW is the one in Fig. 2a). (**d**) The corresponding emissions at Ag NW end for different propagation distances. Dotted curves correspond to the exponentially fitted emission intensity. The scale bar is 5 μm. The error bars show the standard deviations of emission intensities from data collected within about 2 μm ranges.

Instead of moving NP, the excitation fiber tip can be continuously moved along the NW. We have carried out these experiments in a NP-NW structure. Figure 2a shows the selected bright-field optical images of Ag NP-NW structure with NW diameter of 750 nm and length of 41 μm, and Fig. 2b demonstrates the change of emitted intensities at the Ag NW end and NP with the propagation distances respectively. In these experiments, the propagation distance is defined as the distance from the fiber tip to NW end. With increasing the propagation distance, the scattering light at NW end shows an apparent exponenetial decreasing trend with a wavy appearance, unlike the almost steady trend in Fig. 1c. In order to investigate the damping trend precisely, we intentionally remove the NP from the NW, and investigate the emission properties at NW end, as shown in Fig. 2c. The propagation distances are continuously changed when excitation fiber tip is moved along the NW, and the corresponding Ohmic losses vary accordingly. As the NW in Fig. 2b and d is the same individual, exponential damping trend in Fig. 2b and d should be similar to each other. This provides further evidence that the emission trend at NW end in the NP-NW structure arises from the Ohmic loss in NW. When moving tip, the distance between excitation end and NP also changes, and the damping tendency of corresponding propagation loss is consistent with the case in Fig. 1c, resulting in the similar emission intensity trend at NP.

**Figure 3:**
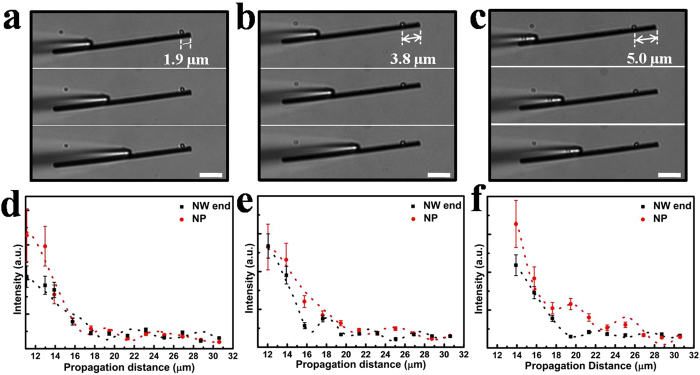
The effect of different positions of Ag NP on the emissions in Ag NP-NW structure. (**a–c**) Bright-field optical images for the Ag NP-NW structure with three different positions of NP (a: 1.9 μm, b: 3.8 μm, c: 5.0 μm). The scale bar is 5 μm. (**d–f**) The corresponding intensities of emission light from Ag NP and Ag NW end to Fig. a–c. Dotted curves correspond to the fitted emission intensity. The error bars show the standard deviations of emission intensities from data collected within about 2 μm ranges.

In order to further investigate the influence of NP position on SPPs propagation in NW, we systematically carried out experiments with different NP locations as shown in Fig. 3. An Ag NW (with diameter of 630 nm and length of 32 μm) with three different positions of Ag NP (the distance between the NP and NW terminal is 1.9 μm, 3.8 μm, 5.0 μm respectively) is employed. Selected bright field optical images are displayed in Fig. 3a–c. Figure 3d–f show the output light intensities at NP and NW end. The emissions at NP and NW end both show exponential damping trend together with undulating behaviour, which are consistent with the results in Fig. 2b. It can be seen that when the distance between NP and NW end is 5.0 μm, the emission intensity at NP is almost the strongest compared with the distance of 1.9 μm and 3.8 μm. This should be related to the relative strong local SPPs field strength at the distance of 5.0 μm, which can cause more SPPs scattered out from the NW. Therefore, the position of NP is an important factor for tuning the behaviour of scattering light in NP-NW structures. Moreover, it is found that the propagation loss increases with increasing the distance between NP and NW end, and the propagation loss here are all larger than that of the initial NW without NP. For the NW without NP, the propagation loss per unit length along the Ag NW is about 0.44 dB/μm, while for NW with NP, the propagation loss is 0.62 dB/μm, 0.91 dB/μm, 0.95 dB/μm respectively with decreasing the distance between NP and excitation end (Fig. 3a–c). These results are significant for designing SPP-based nanophotonic circuits, where decreasing propagation loss and tuning SPPs propagation are two important factors that should be considered.

**Figure 4:**
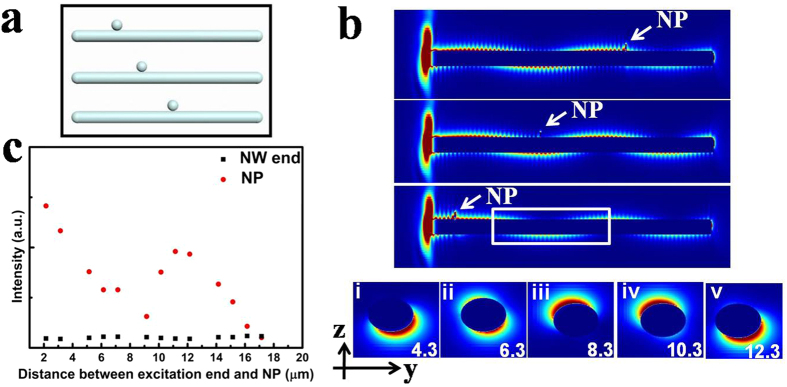
FDTD simulations of the emission in Ag NP-NW structure with continuously tuning NP locations. (**a**) Schematic diagram of the position changes of NP in NW structure. (**b**) Local electric field intensity distributions in NW with three different positions of NP. The wavelength of excitation light is 632.8 nm. The distribution is on the horizontal section across the axis of the NWs. Electric field distribution in the yz plane at different positions along the NP-NW geometry, where x = 4.3 μm to 12.3 μm (i–v) in steps of 2 μm, indicated by the white frame in Fig. b. (**c**) The emission intensities at NP and NW terminal with increasing the distance between the center of NP and the excitation end.

We have performed finite-difference time-domain (FDTD) simulations to examine the emission in NP-NW structure. Figure 4a shows the schematic diagram of NP-NW structure with continuously tuning NP locations in simulation. Figure 4b illustrates local electric field intensity distributions in NW with three different positions of NP and SPPs field distribution in the yz plane along the NP-NW structure. When the polarization angle of the excitation light is 45°, the primary modes are fundamental modes, including TM_0_, 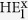 and 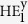. The interference between these modes and the counter propagating SPP modes can form the beat SPPs field distribution along the NW. As indicated by the white frame in Fig. 4b, the electric field distribution of the SPPs in the yz plane at different positions along the NP-NW geometry, where x = 4.3 μm to 12.3 μm (i–v) in steps of 2 μm, directly reflect the beat distribution of SPPs field. When increasing the distance between NP and excitation end, emission intensity at NP shows an exponential decay with a large wavy pattern. The oscillating period is about 10 μm, which is larger than the experimental results. It is probably related to reflection efficiency of SPP modes from the NW end and the excitation of SPP modes as well as the polarization of excitation light. The emission at NW end is nearly a constant, which seems to be rarely affected by the NP. There might be two reasons. On the one hand, the emission intensity at NW end mainly depends on the Ohmic loss in NW, which is almost kept constant due to the unchanged distance. On the other hand, the NW end is located at the node of the beat field distribution, just like the distal of an oscillating rope when fixed. Thus, the emission intensity at NW end is relatively weak and probably rarely affected by the emitted light at NP. These simulation results are in good agreement with our experimental results as shown in Fig. 1c, although the wavy amplitude of the emission at NP is larger and the emission at NW end is nearly unchanged in the simulation results.

## Conclusions

In summary, we experimentally investigate the SPPs propagation properties through continuously moving an Ag NP along a NW and demonstrate the significant role of NP location on influencing the SPPs propagation in NW. Through changing the position of Ag NP, we find that the emission intensity at Ag NP shows an exponential decay together with an undulating pattern, while the emission at NW end vibrates around a constant value. We find that the emission at NP is related to the local SPPs field strength in NW and the emission at NW end are mainly dependent on the distance between the excitation end and the NW terminal. Moreover, we systematically investigate the effect of NP location on the propagating SPPs in Ag NW waveguide, and find that the propagation loss in NW with NP becomes larger than the case in the same NW without NP. These results are helpful for the future photonic circuits and devices. Our work also highlights the significance of choosing proper position of symmetry breaking in structural geometry for designing and achieving high-performance nanophotonic devices.

## Additional Information

**How to cite this article**: Wu, F. *et al*. Control on Surface Plasmon Polaritons Propagation Properties by Continuously Moving a Nanoparticle along a Silver Nanowire Waveguide. *Sci. Rep*. **6**, 37512; doi: 10.1038/srep37512 (2016).

**Publisher’s note:** Springer Nature remains neutral with regard to jurisdictional claims in published maps and institutional affiliations.
